# What is the clinical relevance of different lung compartments?

**DOI:** 10.1186/1471-2466-9-39

**Published:** 2009-08-11

**Authors:** Thomas Tschernig, Reinhard Pabst

**Affiliations:** 1 Functional and Applied Anatomy, Medical School of Hannover, Carl-Neuberg-Str. 1, 30625 Hannover, Germany

## Abstract

The lung consists of at least seven compartments with relevance to immune reactions. Compartment 1 - the bronchoalveolar lavage (BAL), which represents the cells of the bronchoalveolar space: From a diagnostic point of view the bronchoalveolar space is the most important because it is easily accessible in laboratory animals, as well as in patients, using BAL. Although this technique has been used for several decades it is still unclear to what extent the BAL represents changes in other lung compartments. Compartment 2 - bronchus-associated lymphoid tissue (BALT): In the healthy, BALT can be found only in childhood. The role of BALT in the development of the mucosal immunity of the pulmonary surfaces has not yet been resolved. However, it might be an important tool for inhalative vaccination strategies. Compartment 3 - conducting airway mucosa: A third compartment is the bronchial epithelium and the submucosa, which both contain a distinct pool of leukocytes (e.g. intraepithelial lymphocytes, IEL). This again is also accessible via bronchoscopy. Compartment 4 - draining lymph nodes/Compartment 5 - lung parenchyma: Transbronchial biopsies are more difficult to perform but provide access to two additional compartments - lymph nodes with the draining lymphatics and lung parenchyma, which roughly means "interstitial" lung tissue. Compartment 6 - the intravascular leukocyte pool: The intravascular compartment lies between the systemic circulation and inflamed lung compartments. Compartment 7 - periarterial space: Finally, there is a unique, lung-specific space around the pulmonary arteries which contains blood and lymph capillaries. There are indications that this "periarterial space" may be involved in the pulmonary host defense.

All these compartments are connected but the functional network is not yet fully understood. A better knowledge of the complex interactions could improve diagnosis and therapy, or enable preventive approaches of local immunization.

## Commentary

**Figure 1:**
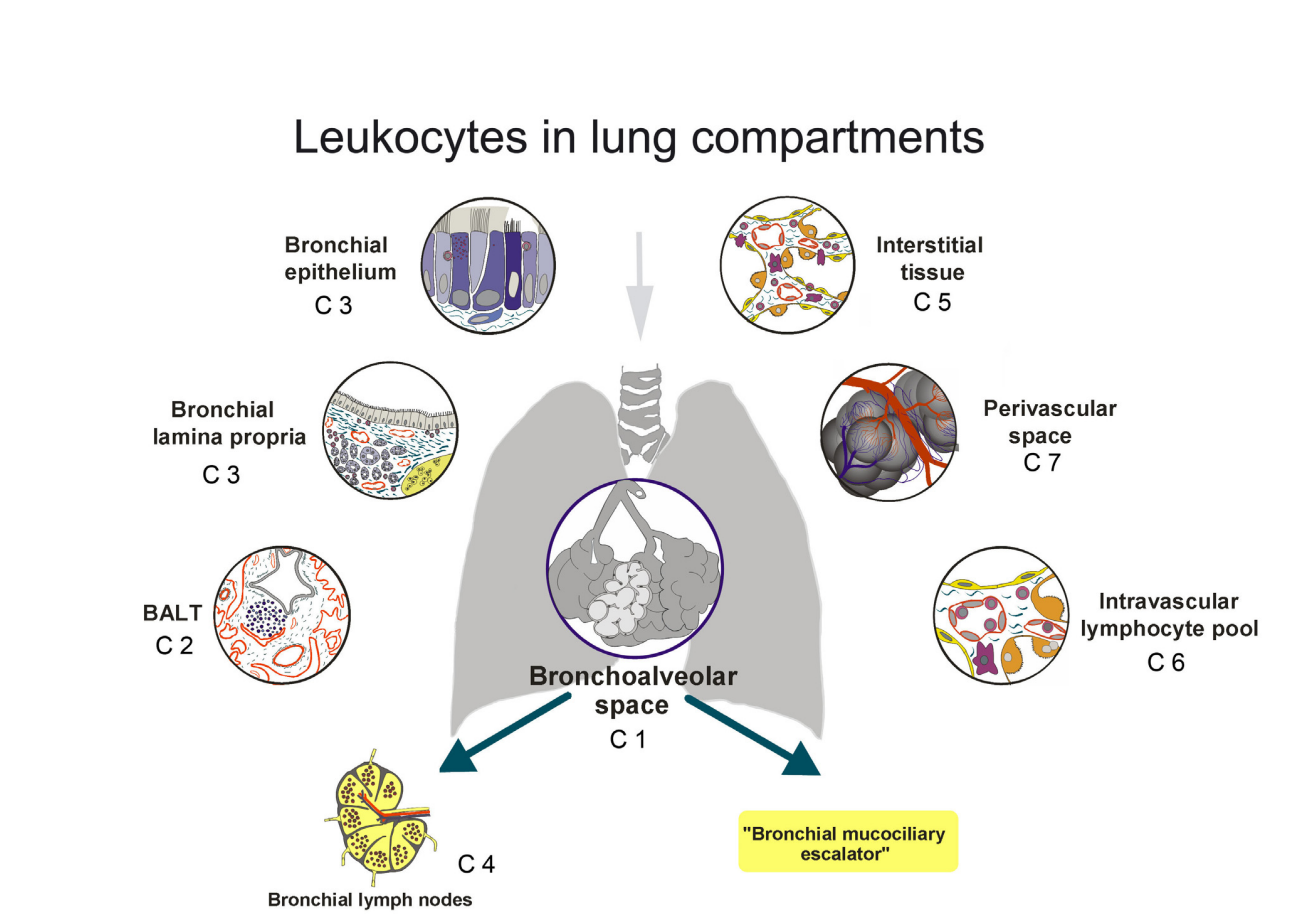
**Schematic drawing of the different lung compartments and exit routes**. The figure was modified after Pabst and Tschernig, 1997 [2].

The lung is a complex organ and anatomically or functionally it can be divided into different cellular compartments such as the bronchoalveolar space, bronchus-associated lymphoid tissue (BALT) and the bronchial mucosa, the lung parenchyma or interstitium, the draining lymph nodes, the intravascular cell pool and the periarterial space (Figure 1) [1]. The aim of this review is first to give an overview about the different lung compartments. It is very important to separate "the lung" into those functionally different subunits to understand the development and the course of immune responses and inflammatory diseases within this organ. Furthermore, a second aim is to point out open questions on the specific role of those compartments. Finally, a concept will be proposed on "master compartments" and the most urgent questions to be resolved.

### Compartment 1 - BAL

The bronchoalveolar lavage (**BAL**) is the most frequently used diagnostic method to gain insight into the lung, yielding leukocytes, secretions, and epithelial cells from the alveoli and the airway mucosa. From experience a number of diagnoses are supported by a specific cytology, for example in asthma, infections, sarcoidosis, tumors [2,3]. But do those cells reflect the pathophysiology in other lung compartments? Moreover, how do the leukocytes arrive in the bronchoalveolar space and what is their role there? This is easy to answer in infection when neutrophils and exsudate macrophages combat bacilli or virally infected cells, but it is unclear in asthma or sarcoidosis. Induced sputum yields cells from the same compartment in a modified composition but is used to a much lesser extent. The other direction of leukocyte migration is also possible: It has been documented in pigs and rodents that lymphocytes can return to the lung interstitium and then reach the regional lymph nodes [4]. Comparable data have been obtained for other immune cells, e.g. dendritic cells.

*The use of BAL has been documented to be very helpful in clinical diagnosis and in experimental models of lung diseases. However, there are still several unresolved issues regarding leucocytes and other immunological cells in the bronchoalveolar space. Is the influx of leukocytes in asthma or sarcoidosis functional or just a spill over? Does the differential of lymphocytes reflect the composition in the interstitium or the intravascular space? Is the reentry of dendritic cells, macrophages and lymphocytes important for the maintenance of the disease? These issues will need to be addressed in further research. Regarding the first two issues a comparison of gene and protein expression of the separate compartments would be useful. The last question might be addressed using experiments blocking the reentry of leukocytes*.

### Compartment 2 - BALT

**BALT **is absent in adults but can be found in about 40% of children [5]. Under some conditions (inflammation, smoking) it can reappear, but it remains totally unclear what that means for disease [6]. From animal studies it is known that BALT is part of the integrated mucosal immune system [7]. This means that lymphoblasts and lymphocytes migrate from BALT to other mucosal sites. A new interesting approach could be to induce BALT, as has been successfully performed in rats by a single intratracheal application of the lipopeptide MALP-2 [8] in order to efficiently apply drugs and vaccines to the lung.

*Prior to this many open questions still have to be answered. The role of BALT is not yet known: What is the stimulus and the mode of forming BALT "de novo" in certain clinical conditions? Is newly induced BALT good or bad for the patient? Could BALT be induced and serve in the delivery of drugs and vaccines? The last question should be addressed experimentally and might lead to new therapeutic strategies in future*.

### Compartment 3 - conducting airway mucosa

The **IEL **and **submucosal leukocytes **are of great relevance in allergic inflammation and bronchial infections. They have many functions in the onset and perpetuation, for example, of asthma [9]. The numbers of dendritic cells have recently been determined in the human tracheal mucosa [10]. It is known that mucosal leukocytes crosstalk with the bronchial epithelium in down- or up-regulation of inflammatory processes [11]. However, the functional network of this leukocyte pool and its subset composition are not yet fully understood. Since the bronchial circulation is the basis for these cells, major species differences have to be considered: In contrast to the well vascularized bronchial mucosa in humans, the mouse lacks a bronchial circulation [12]. This is very important in the interpretation and comparison of experimental data.

Some questions have to be addressed in further experimental research in vitro and in vivo. Who is in the driving seat, the epithelium or the leukocytes? A research approach could be the transfer of leukocytes to SCID mice and subsequent infection. Are regional differences from the trachea to small bronchi of clinical relevance? What is the effect of different subset compositions of the epithelium in different segments of the air conducting parts?

### Compartment 4 - draining lymph nodes

The draining lymph vessels and the **draining lymph nodes **are an essential part of the pulmonary immune system. They are important sites for adaptive/protective immune responses in infection and allergy [1].

*Since there are abundant data on the lung draining lymph nodes it is important to clarify the following open questions in the near future: Is there a functional integration of the lung draining lymph nodes in the general immune system and how is a potential crosstalk regulated? In the gut immune response the mesenteric lymph nodes play an important role in tolerance. What is the significance of lymph nodes draining the lung in this respect? Which adhesion molecules regulate the entry of leukocytes from the blood and the release into the efferent lymphatics? To address these questions animals without lymph nodes such as lymphotoxin knock out mice could be used. Blocking strategies of adhesion molecules and chemokine receptors can give insight into the migration of immune cells to the lymphatics*.

### Compartment 5 - lung parenchyma

The **parenchyma **is a mixture of capillaries, pneumocytes and many other cell types [13], within which the **interstitium **can be delineated [1]. It contains the smallest arterioles and venules, initial lymph vessels, macrophages and dendritic cells, fibrocytes/blasts and is the first place for the infiltration of exsudate leukocytes besides the alveoli. Here interstitial inflammation and fibrosis start and continue. The role of this compartment, for example in asthma, remains to be elucidated.

Other questions are: What are the pro- and anti-inflammatory regulators in the interstitial lung tissue? What kind of diagnostic approach would be suitable for the situation in this compartment? What is the role of the parenchyma in asthmatic inflammation in the early and late chronic phase?

### Compartment 6 - intravascular leukocyte pool

The **intravascular pool **of leukocytes adhering to the endothelium of capillaries and venules of the lung is very important for the extent of immune reactions in the lung, but little is known about it [1,16,17]. Furthermore, there are significant species differences, e.g. in the presence of so-called PIMs (pulmonary intravascular macrophages) [18].

*It is still unclear whether the intravascular leukocytes are a prerequisite for efficient immune reactions in the lung and how the numbers of these leukocytes and their emigration into the lung parenchyma is regulated. It will be an important target to increase or decrease those numbers in order to modulate pulmonary infections and inflammatory reactions*.

### Compartment 7 - periarterial space

The **periarterial space **was described a few years ago [14]. A number of lymphatic vessels and a capillary network are located around the big and smallest pulmonary arteries. Very recently its role in pulmonary host defense has been shown [15]. Since this lung compartment is quite "young" it represents a new focus in experimental and clinical research.

*For example, what kind of immunity is dominant in the periarterial space, innate or adaptive immunity? What are the mechanisms directing the infiltrating cells from the alveoli to the periarterial space? Which factors regulate the clearance of the immune cells after the immune reaction has been completed? Experimental methods to address these questions could be the use of in vitro multi photon microscopy or other new live imaging techniques which allow the dynamics of periarterial inflammation to be observed*.

## Concluding remarks

All the presented compartments of the lung are essential for the immune control of the whole organ. However, three compartments need to be studied with priority. These "master compartments" of the lung during immune response or host defense are: A) The intravascular pool of leukocytes. This is because of its interaction with lung vessels and capillaries, which controls the entry of leukocytes into different lung compartments and also the integrity of the alveolo-capillary barrier. B) The bronchial mucosa of the conducting airways, which is the first line of defense if microbes or allergens enter the lung. C) The periarterial space, because periarterial inflammation is an important part of the intrapulmonary immune response and a mechanism which keeps the alveoli and therefore the gas exchange area functional. All lung compartments contribute to the innate and the adaptive immune response. The most important questions might be: How is the influx of leucocytes into lung compartments regulated? Which patterns of migration between those compartments are important for immune control? What are the mechanisms for the initiation of perivascular inflammation and -at least equally important - its cessation and how could all these processes be blocked or stimulated by drugs?

## Competing interests

The authors declare that they have no competing interests.

## Authors' contributions

TT and RP have worked together for many years in research on the host defense of the lung, and have continuously discussed morphological issues of the lung and their functional relevance.

## Pre-publication history

The pre-publication history for this paper can be accessed here:

http://www.biomedcentral.com/1471-2466/9/39/prepub

## References

[B1] Pabst R Tschernig T Lymphocytes in the lung: an often neglected cell. Numbers, characterization and compartmentalization Anat Embryol 1995 192 293 9 10.1007/BF00710098 8554162

[B2] Pabst R Tschernig T Lymphocyte dynamics: caution in interpreting BAL numbers Thorax 1997 52 1078 80 9516903 10.1136/thx.52.12.1078 PMC1758469

[B3] Meyer KC Bronchoalveolar lavage as a diagnostic tool Semin Respir Crit Care Med 2007 28 546 60 10.1055/s-2007-991527 17975782

[B4] Lehmann C Wilkening A Leiber D Markus A Krug N Pabst R Tschernig T Lymphocytes in the bronchoalveolar space reenter the lung tissue by means of the alveolar epithelium, migrate to regional lymph nodes, and subsequently rejoin the systemic immune system Anat Rec 2001 264 229 36 10.1002/ar.1163 11596005

[B5] Tschernig T Kleemann WJ Pabst R Bronchus-associated lymphoid tissue (BALT) in the lungs of children who had died from sudden infant death syndrome and other causes Thorax 1995 50 658 60 10.1136/thx.50.6.658 7638809 PMC1021267

[B6] Tschernig T Pabst R Bronchus-associated lymphoid tissue (BALT) is not present in the normal adult lung but in different diseases Pathobiology 2000 68 1 8 10.1159/000028109 10859525

[B7] Brandtzaeg P Pabst R Let's go mucosal: communication on slippery ground Trends Immunol 2004 25 570 7 10.1016/j.it.2004.09.005 15489184

[B8] Pabst R Durak D Roos A Lührmann A Tschernig T TLR2/6 stimulation of the rat lung: effects on lymphocyte subsets, natural killer cells and dendritic cells in different parts of the air-conducting compartments and at different ages Immunology 2009 126 132 9 10.1111/j.1365-2567.2008.02886.x 18565128 PMC2632703

[B9] Synek M Beasley R Frew AJ Goulding D Holloway L Lampe FC Roche WR Holgate ST Cellular infiltration of the airways in asthma of varying severity Am J Respir Crit Care Med 1996 154 224 30 8680684 10.1164/ajrccm.154.1.8680684

[B10] Tschernig T de Vries VC Debertin AS Braun A Walles T Traub F Pabst R Density of dendritic cells in the human tracheal mucosa is age dependent and site specific Thorax 2006 61 986 91 10.1136/thx.2006.060335 16893947 PMC2121158

[B11] van Tongeren J Reinartz SM Fokkens WJ de Jong EC van Drunen CM Interactions between epithelial cells and dendritic cells in airway immune responses: lessons from allergic airway disease Allergy 2008 63 1124 35 10.1111/j.1398-9995.2008.01791.x 18699930

[B12] Tschernig T Neumann D Pich A Dorsch M Pabst R Experimental bronchial asthma - the strength of the species rat Curr Drug Targets 2008 9 466 9 10.2174/138945008784533543 18537585

[B13] Burri PH Morphology and respiratory function of the alveolar unit Int Arch Allergy Appl Immunol 1985 76 S1 2 12 10.1159/000233728 3838533

[B14] Pabst R Tschernig T Perivascular capillaries in the lung: an important but neglected vascular bed in immune reactions? J Allergy Clin Immunol 2002 110 209 14 10.1067/mai.2002.126836 12170259

[B15] Reppe K Tschernig T Lührmann A van Laak V Grote K Zemlin MV Gutbier B Müller HC Kursar M Schütte H Rosseau S Pabst R Suttorp N Witzenrath M Immunostimulation with macrophage-activating lipopeptide-2 increased survival in murine pneumonia Am J Respir Cell Mol Biol 2009 40 474 81 10.1165/rcmb.2008-0071OC 18931326

[B16] Gill SS Suri SS Janardhan KS Caldwell S Duke T Singh B Role of pulmonary intravascular macrophages in endotoxin-induced lung inflammation and mortality in a rat model Respir Res 2008 9 69 10.1186/1465-9921-9-69 18950499 PMC2584635

[B17] Winkler GC Review of the significance of pulmonary intravascular macrophages with respect to animal species and age Exp Cell Biol 1989 57 281 6 2519957 10.1159/000163539

[B18] Warner AE Brain JD The cell biology and pathogenic role of pulmonary intravascular macrophages Am J Physiol 1990 258 L1 12 2407136 10.1152/ajplung.1990.258.2.L1

